# Evaluation of Phenobarbital Adsorption Efficiency on Biosorbents or Activated Carbon Obtained from Adansonia Digitata Shells

**DOI:** 10.3390/ma17071591

**Published:** 2024-03-30

**Authors:** Charnella Stevine Dibandjo Ndankou, Daniela Simina Ștefan, Ndi Julius Nsami, Kouotou Daouda, Magdalena Bosomoiu

**Affiliations:** 1 Faculty of Chemical Engineering and Biotechnology, National University of Science and Technology Politehnica of Bucharest, 1-7 Gheorghe Polizu Street, 011061 Bucharest, Romania; stevine.dibandjo@facsciences-uy1.cm (C.S.D.N.); daniela.stefan@upb.ro (D.S.Ș.); 2 Applied Physical and Analytical Chemistry Laboratory, Department of Inorganic Chemistry, Faculty of Science, University of Yaoundé I, Yaoundé P.O. Box 812, Cameroon; julius.ndi@facsciences-uy1.cm (N.J.N.); daouda.kouotou@facsciences-uy1.cm (K.D.)

**Keywords:** adsorption, phenobarbital, biosorbents, activated carbon, adsorption isotherms, particle size, adsorption kinetics

## Abstract

The removal of pharmaceutically active compounds present in relatively low concentration in wastewater is critical. This is because they have a severe, negative impact on life and the environment. To address this issue, adsorption was used, which is an effective wastewater treatment method for removing substances found in low concentrations in water. This study compared the adsorption performance of active carbon to three biosorbents derived from Adansonia digitata shells. The adsorbents were prepared and characterized using TGA, SEM, EDX, and FTIR analyses and pH_PZC_. To better understand the adsorption process, equilibrium and reaction kinetics studies were conducted. The effect of contact time, initial phenobarbital concentration, adsorbent mass, and pH was investigated in static conditions. The adsorption results revealed that the biosorbent B3 has a higher affinity for the eliminated compound, with an equilibrium time of 60 min and an adsorption capacity of 47.08 mg/g at an initial concentration of 50 mg/L. The experimental data are consistent with Langmuir and Sips adsorption isotherm models, and with the pseudo-second order and Elovich models for kinetics description. This indicates strong interactions between the adsorbent materials and the pharmaceutical micropollutant. Based on these findings, it appears that, among the tested materials, B3 biosorbent is the most efficient for removing phenobarbital present in low concentrations in water.

## 1. Introduction

Environmental pollution is a major global issue. Water is a vital resource; in the context of accelerated climate change, water resources and their quality are decreasing rapidly. Lack of water resources affects human populations and natural ecosystems and contributes to poverty in countries already experiencing slow development. The deterioration of the quality of water resources is primarily caused by human activities, agricultural practices, and industrial discharges into waterways. These discharges frequently include endocrine disruptors, surfactants, cosmetics, and active pharmaceutical ingredients that considerably alter the quality of water resources. The latter, mostly emerging contaminants, are not completely eliminated by wastewater treatment plants and accumulate in the environment, endangering biodiversity [1]. Among these contaminants, phenobarbital (PHB), a barbiturate drug used to treat epilepsy, insomnia, and as a sedative, is especially concerning [2]. It has been found in water and is regarded as a hazardous environmental pollutant [3]. It has, thus, been designated as a high-prevalence aquatic contaminant [4]. Scientific research studies have proposed several water treatment techniques, including advanced oxidation [5], photo degradation [6], electrochemical degradation [7], and the Fenton process [8]. However, these techniques have significant disadvantages, including low efficiency, high implementation costs [9], and the generation of harmful byproducts [10]. Studies performed on samples taken from wastewater treatment plants showed that phenobarbital is among the antiepileptics found in the water evacuated from the treatment plants [11,12,13]. This suggests that the existent technologies are not yet adapted to satisfactorily remove the pharmaceutical compounds from wastewater. Previous research studies have shown that adsorption is a promising method for removing these substances, even at low concentrations [14], due to its high efficiency, ease of use, and the regenerability of the adsorbent, which limits the quantity of byproducts [15]. It is a surface process in which the adsorbate and the adsorbent interact without affecting the latter’s properties. Several adsorbents have been used over the years, but due to their poor performance in highly polluted environments, researchers have introduced carbonaceous (activated carbons) and non-carbonaceous (biosorbents) adsorbents derived from algae, wood, and agricultural by-products. Because of its availability and high adsorption capacity, activated carbon has received extensive research as an adsorbent in wastewater treatment. Another type of effective adsorption materials are biosorbents, which have a high capacity to adsorb organic compounds. Their adsorption capacity is due to the presence of functional groups like hydroxyl, carboxyl, and amine groups, as well as their large specific surface area and porosity [16]. These materials were found to have a high adsorption potential for heavy metals [17], dyes [18], and organic contaminants [19]. Consequently, it is necessary to develop low-cost, environmentally friendly adsorbent materials from renewable organic resources, such as agricultural by-products, in order to set up efficient, sustainable wastewater treatment technologies. The baobab, scientifically known as Adansonia digitata, is a tree found in the dry, hot savannahs of Australia, and Africa. It belonged to the Bombaceae family [20]. Obtaining baobab seeds, which are used by most people for medicine, food, and drink [21], generates massive amounts of waste shells.

According to the data published by FAO (Food and Agriculture Organization), Cameroon is harvesting a surface of about 760 ha annually (data valid for 2021 and 2022), cultivated with trees producing fruits in shells [22]. The quantity of baobab shells released depends on local production and fruit consumption. In the context of the circular economy concept, it is desired to valorise these residues by using them as adsorbent materials for the advanced elimination of some dangerous organic compounds from the wastewater.

Studies have been conducted to characterise these shells, and the findings indicate that their properties are particularly promising. For example, baobab shells contained 54.08% lignin, 24.87% cellulose, 21.05% hemicellulose, 5.17% ash, 6.48% moisture, and 1.22% carbon [23]. These unique properties make baobab hulls promising adsorbents for wastewater treatment [23].

Although much work has been done on the removal of emerging contaminants from water, the waste generated by baobab shells drew our attention due to their potential as a good candidate as adsorbent materials. In our study, we present materials (biosorbents obtained without chemical treatment and activated carbon) manufactured from Adansonia digitata (AD) hulls used to remove phenobarbital (PHB) in aqueous solution.

The goal of this research is to demonstrate the preparation of biosorbents of various sizes and activated carbon based on AD shells, as well as to evaluate their effectiveness against the PHB removal from water. The biosorbents used were freshly harvested and collected for free in northern Cameroon, notably from the Adamaoua region (Ngaoundéré). The characterization-employed techniques include Fourier transform infrared spectroscopy (FTIR), scanning electron microscopy coupled to energy dispersive X-ray spectroscopy (SEM/EDX), thermogravimetric analysis (TGA), and zero-charge point pH (pH_PZC_). Batch experiments were conducted to assess the adsorption capacities, isotherms, and kinetics of PHB adsorption on the prepared materials. An adsorption mechanism was suggested. By using agricultural waste materials, available at a relatively reduced cost and environmentally friendly, we are minimizing waste production and contributing to sustainable agriculture.

To our knowledge, this study represents a novelty. We are presenting here, for the first time, materials derived from Adansonia digitata (AD) shells as adsorption materials used to eliminate phenobarbital from aqueous solutions.

## 2. Materials and Methods

### 2.1. Chemicals and Reagents

All chemicals were used without further purification. Phenobarbital (C_12_H_12_N_2_O_3_, 99%), natrium chloride (NaCl) and sodium hydroxide (NaOH) were purchased from Sigma–Aldrich (St. Louis, MO, USA); borax (Na_2_B_4_O_7_), and ammonium buffer solution were purchased from Merck (Rahway, NJ, USA); ethanol (C_2_H_6_O, 99%), hydrochloric acid (HCl) and phosphoric acid (H_3_PO_4_, 85%) were purchased from Riedel–de Haën (Seelze, Germany). All the solution were prepared using deionized water. Adansonia digitata shells were collected in northern Cameroon, more specifically, in the Adamaoua region (Ngaoundéré), and were washed with tap water to remove dirt.

### 2.2. Preparation of Adsorbents

BIOSORBENTS

The AD shells were crushed and sieved into various fractions (greater than 1.6 mm, greater than 0.16 mm and less than 0.16 mm). The particles were washed with distilled water and oven-dried for 3 h at 110 °C. The obtained materials were labelled as B1, B2 and B3, corresponding to each fraction, respectively.

AC ADSORBENT

Chemical activation was used to produce activated carbon, with phosphoric acid serving as the activating agent. In total, 10.0 g of dried and ground AD shells were impregnated with 6.3 mL of H_3_PO_4_ solution at the ratio 1:1. The mixture was left at room temperature for one hour to allow the reagents to be completely absorbed into the AD shells network. The impregnated AD shells were dried in an oven at 110 °C for 2 h, then cooled in a desiccator before being carbonized in a muffle furnace (Carbolite Furnace, Sheffield, UK) at 332 °C for 1 h and 37 min at a heating rate of 10 °C/min. We obtained the best conditions for the preparation of activated carbon using the Minitab software, version 18 applying the design of experiments methodology and, more specifically, using the central composite design. After furnace cooled down to ambient temperature, the obtained activated carbon was collected and washed with deionized water until neutral pH was obtained. The resulting material was labelled as being (AC).

### 2.3. Preparation of the Phenobarbital Solution

A total of 1000 mg/L of the stock solution of phenobarbital (Table 1) was prepared by dissolving 1000 mg of pure phenobarbital powder in a mixture of deionized water and ethanol in a ratio of 49:1 (980 mL of deionized water and 10 mL of ethanol). The working solutions with concentrations ranging from 1 mg/L to 250 mg/L were prepared by suitable dilution.

### 2.4. Samples Characterisation

Various approaches were used to characterize the obtained samples. Thermogravimetric analysis data (TGA) coupled with Differential Scanning Calorimetric (DSC) was recorded on a TGA/-IST instrument (Thermal Analysis System TGA 2, METTLER TOLEDO, Greifensee, Switzerland), by heating the samples in an aluminium oxide crucible from 25 to 900 °C at a rate of 10 °C/min under a 50 mL/min nitrogen flow. The chemical properties of the adsorbents were examined under Fourier transform infrared spectroscopy (FTIR) using a Nicolet iS50FT-IR (Nicolet, Dracut, MA, USA) spectrophotometer, equipped with a DTGS detector which has a high sensitivity in the range of 4000 cm^−1^ and 100 cm^−1^. The FTIR spectra of ATR pellets of the prepared materials were recorded using a FTIR spectrophotometer in absorbance mode with a resolution of 4 cm^−1^ to determine their surface functional groups. Each of the adsorbents were analysed using a scanning electron microscope (Quanta INSPECT F50, FEI Company, Eindhoven, The Netherlands) with an accuracy of 1.2 nm and an energy dispersive X-ray spectrometer (EDX) with a resolution of MnK of 133 Ev.

### 2.5. Adsorption Experiments

All adsorption experiments were carried out at 20 ± 0.6 °C, and the pH of the phenobarbital solution was adjusted from 1 to 9 using ammonia buffer. In total, 25 mL of a 50 mg/L phenobarbital solution at the desired pH was mixed with 25 mg of the adsorbent. The effect of the adsorbent dose was studied by varying the adsorbent mass in the range of 0.025 to 0.25 g. The mixtures were stirred on a multi-stage stirrer (ORBITAL SHAKER) for 60 min until equilibrium was reached. The suspension was then filtered through filter paper and diluted with borax buffer solution to obtain a pH of 9; for a dilution factor of 5, the phenobarbital concentration was then determined by UV–vis spectrophotometry with a UV-1900 spectrophotometer (Shimadzu, Kyoto, Japan) at λ = 275 nm. Phenobarbital adsorption isotherms on different adsorbents were determined by varying the concentration in the range of 1 to 250 mg/L. Similarly, the adsorption kinetics of phenobarbital on the different adsorbents were determined at different times in the range of 1 to 240 min. The quantity adsorbed at equilibrium and at time t was calculated according to Equations (1) and (2), respectively:

(1) $$q_{e}=\frac{\left(C_{0}-C_{e}\right)\times V}{m}$$

(2) $$q_{t}=\frac{\left(C_{0}-C_{t}\right)\times V}{m}$$

where, C_0_, C_e_ and C_t_ represent the concentration of phenobarbital (mg/L) at the initial stage, equilibrium and at any time, respectively, V is the volume of the solution, and m is the mass of adsorbent sampled.

## 3. Results and Discussions

### 3.1. Thermogravimetric Analysis of Adsorbent Materials

**Figure 1:**
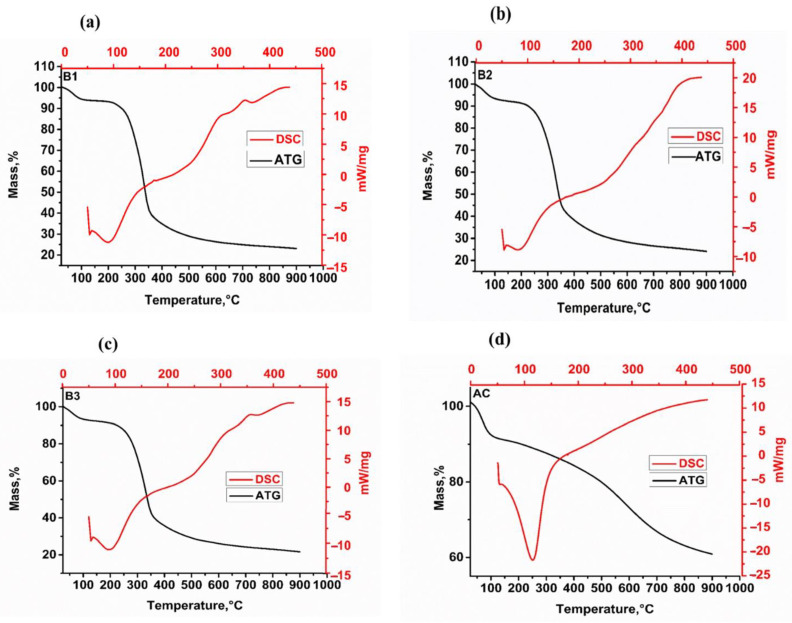
TGA and DSC of biosorbents (**a**)—B1, (**b**)—B2, (**c**)—B3 and the activated carbon (**d**)—(AC).

Figure 1 depicts the TGA and DSC profiles of biosorbents and activated carbon. The TGA curves show two stages of weight loss over a temperature range of 25 to 900 °C in an inert atmosphere. The biosorbents experience an initial mass loss of 5.70%, 6.78%, and 7.80% for B1, B2, and B3 at temperatures ranging from 25 to 110 °C. For the AC, the initial mass loss is 9.55% in the temperature range of 25 to 100 °C. This mass loss can be attributed to the loss of free water at the biomass surface and the onset of biomass decomposition, as indicated by the endothermic peak in the DSC profile.

The main mass loss observed between 200 and 475 °C (B1), 200 and 484 °C (B2) and 200 and 378 °C (B3) can be attributed to the degradation of cellulose and one part of the lignin. Because this process generates heat, it is accompanied by an exothermic reaction observed on the DSC curves [25,26]. As a result, we can see that biosorbent mass loss is proportional to particle size; as the particle size decreases, mass loss increases, which can be explained by the fact that the finer the particle, the better the heat transfer, which also improves its reactivity [27]. For activated carbon, significant mass loss occurred in the temperature range of 95 to 846 °C; however, the percentage of mass loss decreases as the pyrolysis temperature increases, as evidenced by an endothermic peak on the DSC. Mass loss above 95 °C (9%) is due to the degradation and decomposition of lignin, which is still present in the material because the pyrolysis temperature used to obtain activated carbon was only 332 °C. Furthermore, mass loss can be linked to the degradation of inorganic species such as CaCO_3_, K_2_CO_3_, and MgCO_3_ [28]. Activated carbon has the highest rate of mass loss of all the materials, so we can conclude that it has the greatest reactivity.

### 3.2. Fourier Transform Infrared Spectroscopy

**Figure 2:**
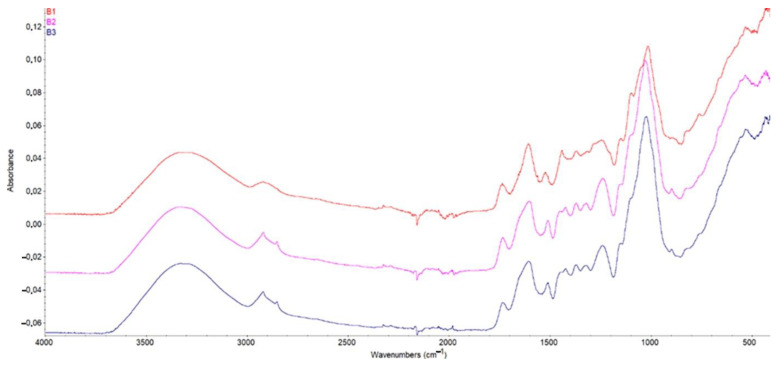
FTIR spectra of biosorbents B1, B2 and B3.

**Figure 3:**
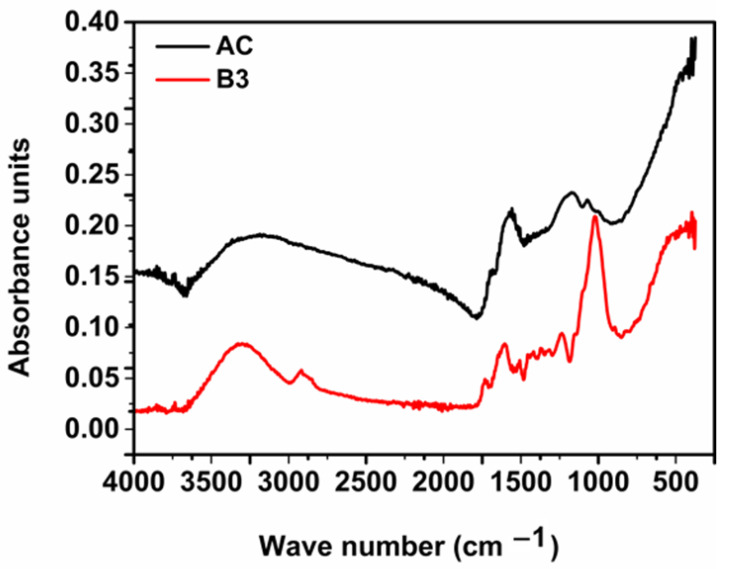
FTIR spectra of activated carbon AC and biosorbent B3.

Figure 2 and Figure 3 show the FTIR spectra of biosorbents B1, B2 and B3 (Figure 2) and of B3 and AC (Figure 3). Given the similarities presented in the literature, the following functional groups can be identified from the characteristic peaks, representing the adsorption bands typical for AD shells.

The peaks at 3300 cm^−1^ (biosorbents) and 3205 cm^−1^ (AC) correspond to hydroxyl group –OH (associated with alcohol or phenol). The presence of hydroxyl groups indicate that the adsorbents can adsorb polar organic compounds or compounds with N-H bonds associated with amines. The adsorption band at 2916 cm^−1^ (biosorbents) corresponds to an absorption band caused by the vibrations of methyl groups (C–H) in organic molecules. This could imply the presence of alkylated compounds in baobab shells. Peaks at 1730 cm^−1^ (biosorbents) correspond to an absorption band characteristic of carbonyl bonds (C=O) found in ketones, aldehydes, and esters. This suggests that there are carbonyl-containing compounds in baobab hulls. Peaks at 1604 cm^−1^ (biosorbents) are typically attributed to the vibrations of aromatic groups (C=C) found in phenolic compounds or aromatic heterocycles. This could imply the presence of aromatic compounds in baobab husks. Peaks at 1507 cm^−1^ (biosorbents) and 1577 cm^−1^ (AC) correspond to the vibrations of aromatic groups, confirming the presence of aromatic compounds in baobab shells. These groups may contribute to the activated carbon’s adsorption capacity for aromatic compounds. The bands at 1243 cm^−1^ (biosorbents) and 1181 cm^−1^ (AC) are commonly associated with C–O group vibrations (ether or alcohol bonds). These groups may be linked to compounds like alcohols, ketones, esters, or ethers. The peaks at 1013 cm^−1^ could indicate vibrations of a C–O–C group (ether bond) or C–OH (alcohol group). The 428 cm^−1^ peaks are generally associated with vibrations of C–C bonds in carbon chains or C–Cl vibrations in halides [29]. The prepared activated carbon has less chemical function than biosorbents due to carbonization.

### 3.3. SEM and EDX Analysis

**Figure 4:**
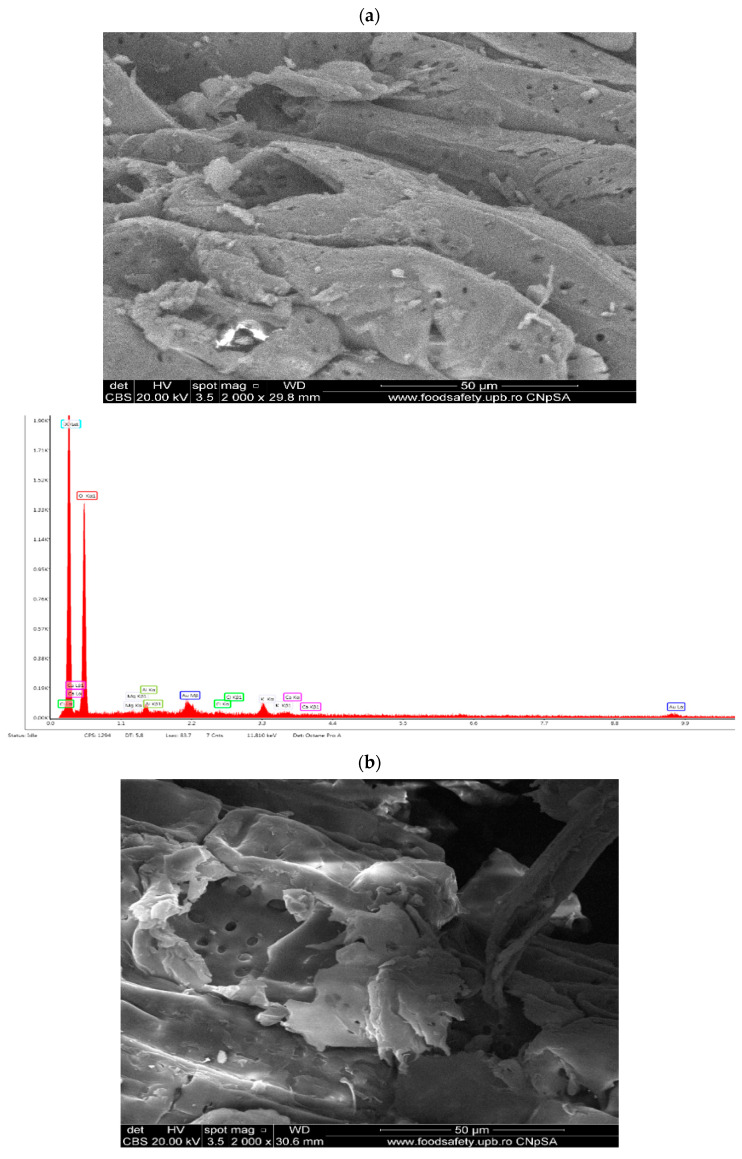

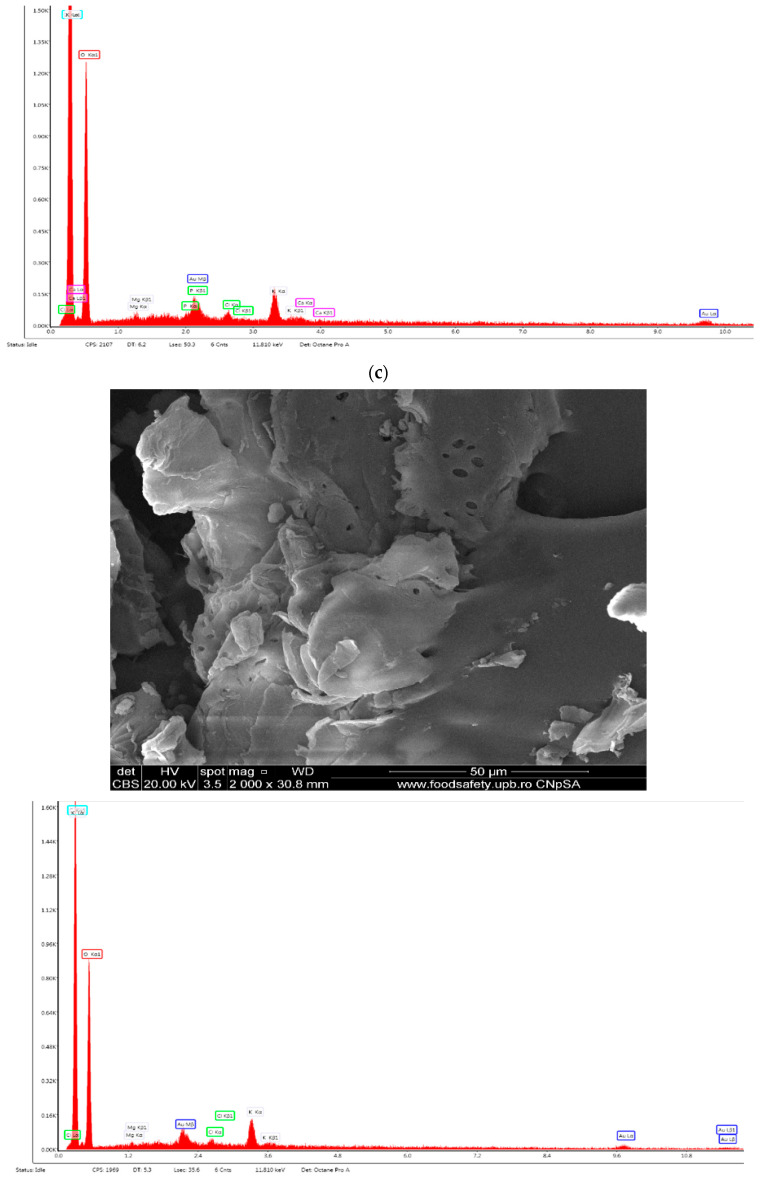

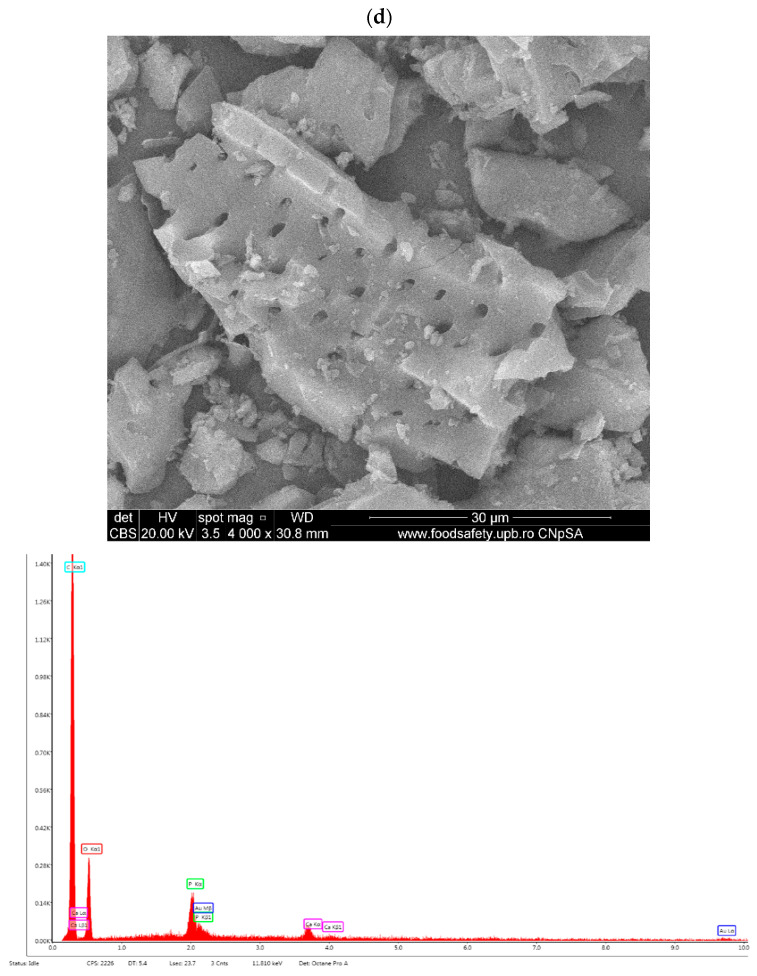
SEM of biosorbents B1 diameters ˃1.6 mm (**a**); B2 diameters ˃ to 0.16 mm (**b**); B3 diameters ˂ to 0.16 mm (**c**); activated carbon (**d**).

The surface structure morphology of AD shells has been visualized using high-resolution SEM (Figure 4). SEM and EDX analyses of biosorbents derived from AD shells are shown in Figure 4a–c. The images show a complex structure with irregular girth, smooth and criss-cross filaments that form a compact mesh, similar to an asymmetrical box set. The illustration depicts spongy surfaces with irregular textures, routing duct, surface cavities, and well-developed porosity because of the presence of a few randomly distributed pores of various sizes. The adsorption capacity increases with the development of the pore structure. The EDX graphs show that all the samples contain elements such as K, O, Ca, P, Mg, Cl, and P, along with traces of other chemical elements.

Figure 4d depicts the SEM/EDX image of activated carbon formed by the chemical activation of baobab shells with phosphoric acid. The use of phosphoric acid as an activator resulted in the development of porosity. As shown in Figure 4d, the surface appears to be more porous than the raw material. According to the EDX results (Figure 4d), the high percentage of phosphorus present in AC was expected because it was activated with H_3_PO_4_, resulting in the incorporation of phosphorus into the structure of AC. Furthermore, the potassium content decreased until it vanished, in comparison to the biosorbents, allowing the carbon to take its place, and the oxygen content decreased. The decrease is due to the increase in temperature.

### 3.4. pH of Point Zero Charge *(*pH_PZC_*)*

**Figure 5:**
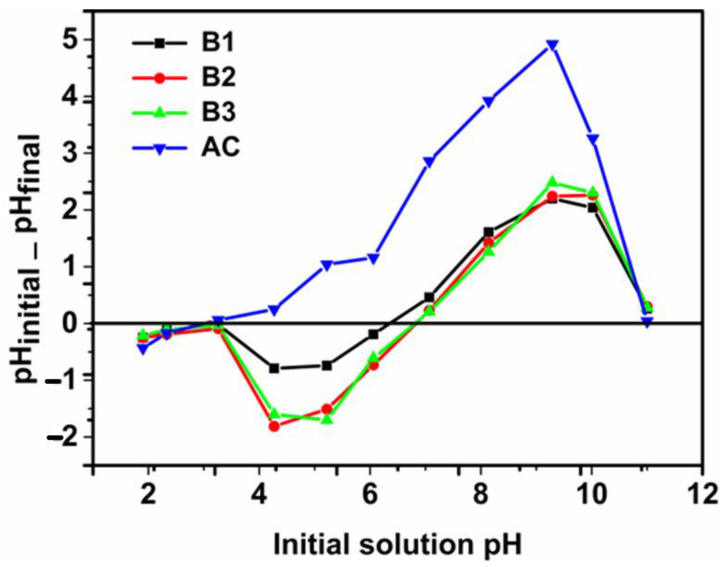
pH of zero charge, pH_PZC_.

The surface charge of the adsorbent is mainly determined by its point of zero charge and the pH of the solution in which it is found. To determine the point of zero charge shown in Figure 5, we followed the protocol proposed by Mehrabi et al. (2015) and Bakatula et al. (2018) [30,31].

The determination of the point of zero charge was carried out using a decimolar solution of NaCl. The pH was adjusted with a 0.1 M solution of NaOH and HCl in the range of 1 to 11. Then, 0.1 g of adsorbents were mixed with 20 mL of solution and stirred for 24 h. The pH of the filtrate was measured and the curve in Figure 5 was obtained (for AC pHpzc = 3.2; for B1 pHpzc = 6.3; for B2 and B3 pHpzc = 6.8). At pH > pHpzc, the surface of our adsorbents is negative and, at pH < pHpzc, it is positive. The pKa value of phenobarbital is 7.4 and, below this value, it remains in neutral form [32].

### 3.5. Phenobarbital Adsorption Tests

**Figure 6:**
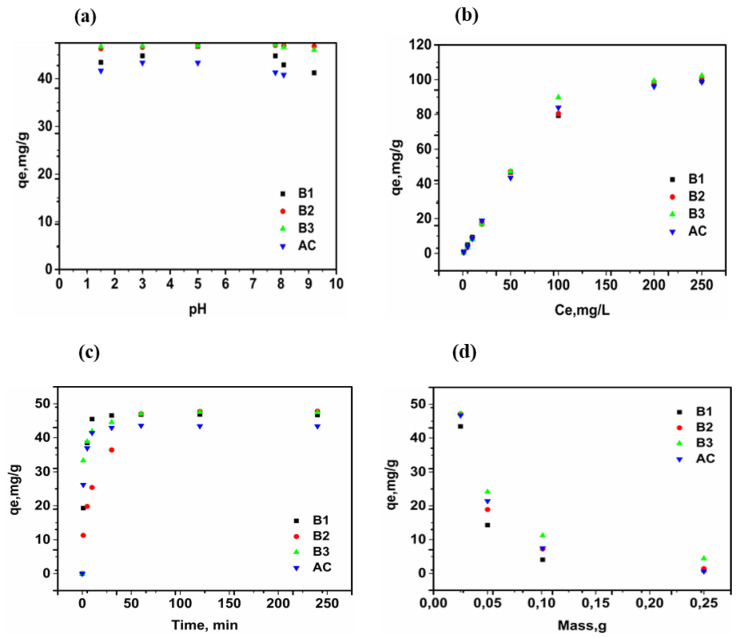
Influence of working conditions: pH, (**a**); Initial concentration, (**b**); Contact time, (**c**); Adsorbent dose, (**d**).

To perform the PHB adsorption study, experiments with different pH solution, PHB initial concentration, contact time, and adsorbent dose were carried out. The results are represented in Figure 6a–d.

#### 3.5.1. Effect of pH Variation

The study of the pH effect is an important step in the adsorption experiments since it involves both the surface functional groups of the adsorbent and the ionization state of the adsorbate. This phenomenon can cause electrostatic attraction or repulsion between the adsorbent and adsorbate [33]. The surface charge of the adsorbent is mainly determined by its point of zero charge and the pH of the solution in which it is found. The adsorbent surface charge is negative at pH > pHpzc and positive at pH < pHpzc, indicating a positively charged surface in an acidic environment. This eliminates the possibility of electrostatic interaction between adsorbents and phenobarbital in acidic media, explaining why the amount adsorbed as a function of pH varies little from point to point. The high values for adsorption capacity indicate the presence of π-π interactions and hydrogen bonds. Adsorption is hampered in basic media due to the electrostatic repulsion between the adsorbent and adsorbate. In the range of the pH values studied, presented in Figure 6a, the quantity of the phenobarbital adsorbed varies little from one point to another. Similar findings were obtained by [33]. This small variation in pH tells us that the adsorption mechanism is not mainly due to electrostatic interactions but rather to the interactions between phenobarbital and the different adsorbents involving π-π interactions and hydrogen bonds. The biosorbents and activated carbon used in this study showed good adsorption capacity for phenobarbital at the various pH values studied. For the remainder of the work, the experiments were carried out at pH 7.4, which is close to the natural pH of phenobarbital.

#### 3.5.2. Effect of Initial PHB Concentration

The effect of the initial concentration was investigated by varying the PHB concentration in the range of 1 to 250 mg/L, using an adsorbent dose of 0.025 g and a contact time of 60 min. The results are depicted in Figure 6b. Increasing the initial concentration of the solution increases the amount adsorbed for all materials until the active sites are nearly saturated. This increase is because, as the concentration of the adsorbate increases, the distance between the phenobarbital molecules decreases, allowing the pollutant to diffuse more rapidly, thereby favouring adsorption.

#### 3.5.3. Effect of Contact Time

The effect of contact time on PHB adsorption was investigated over a range of 1 to 240 min. Solutions were collected at various stirring times, filtered, and the results are shown in Figure 6c. The adsorbed quantity increases in time until equilibrium is reached (rate of adsorption equals rate of desorption). At 10 min, the adsorbed quantities are 45.5, 25.4, 41.9, 41.5 mg/g for B1, B2, B3, and AC, respectively. At 60 min, when the adsorbent surface is fully saturated, the equilibrium adsorbed quantities are 46.8, 47.0, 47.1, 43.6 mg/g, for B1, B2, B3, and AC, respectively. Adsorption capacity was already high within the first 10 min, as demonstrated by [14]. Biosorbent B3 demonstrated a higher adsorption capacity than B1, B2, and AC because it has the smallest particle size and, thus, has the highest specific surface area. Biosorbents’ high affinity for phenobarbital could be attributed to the presence of chemical functions on their surface that are absent on the carbon surface, such as the carbonyl function of aldehydes or ketones, as confirmed by FTIR analysis. Furthermore, the higher PHB adsorption of B3 compared to AC could be explained by the fact that the adsorption process in this case is not only determined by the specific surface area, but also by the interactions between adsorbent and adsorbate.

#### 3.5.4. Effect of Adsorbent Dose

The effect of varying the adsorbent mass on PHB removal was investigated by adjusting the adsorbent mass in the range of 0.025 to 0.25 g. These were mixed with 25 mL of PHB solution; after 60 min, the solutions were filtered, and the filtrate was analysed. Figure 6d shows the amount of PHB adsorbed as a function of adsorbent mass. The adsorption process is influenced by the adsorbent dose; as the adsorbent quantity decreases, the amount of PHB adsorbed per gram of adsorbent increases (m = 0.25 g, q_e_ = 4.394 mg/g; m = 0.025 g, q_e_ = 47.172 mg/g). The maximum adsorption capacity is achieved at a mass of 0.025 g. At higher adsorbent mass, a decrease in the adsorbed quantity per gram of adsorbent was observed; although the higher mass results in a greater number of active sites available for interaction with the adsorbates, the adsorbed quantity tends to decrease. This is due to adsorbent particle agglomeration, which limits the access to the available adsorption sites by increasing the adsorption path [34].

#### 3.5.5. Effect of Particle Size

Particle size is an important factor in determining the adsorption capacity. The effect of particle size on PHB adsorption on various adsorbents was investigated. As explained in the section “Preparation of biosorbents”, the materials B1 to B3 have different particle sizes. The experimental results showed that the amount of PHB adsorbed increases as the particle size decreases. This is explained by the fact that, for the same material, the smaller the particle size, the larger the contact surface, and the adsorption process is intensified [35]. This is the case of biosorbents. However, it should be noted that the activated carbon, having the smallest particle size (0.075 mm), was not the most effective adsorbent. This could be explained by the fact that, in our case, adsorption is determined not only by the adsorbent’s specific surface area, but also by the chemical functions that exist on its surface. The adsorption isotherms confirmed that chemisorption is taking place.

For biosorbents, we conclude that, as the particle size decreases, the amount adsorbed increases because the larger the particle size, the greater the diffusional resistance to mass transfer. Because the path length is longer in bigger particles than in smaller particles, the adsorption capacity of larger particles is lower.

For comparison purposes, the obtained q_e_ values and data reported in the literature for several AC and biosorbents are given in Table 2. It can be seen that the materials we have tested have values higher than other reported biomass-based adsorbents.

### 3.6. Adsorption Kinetics Studies

**Figure 7:**
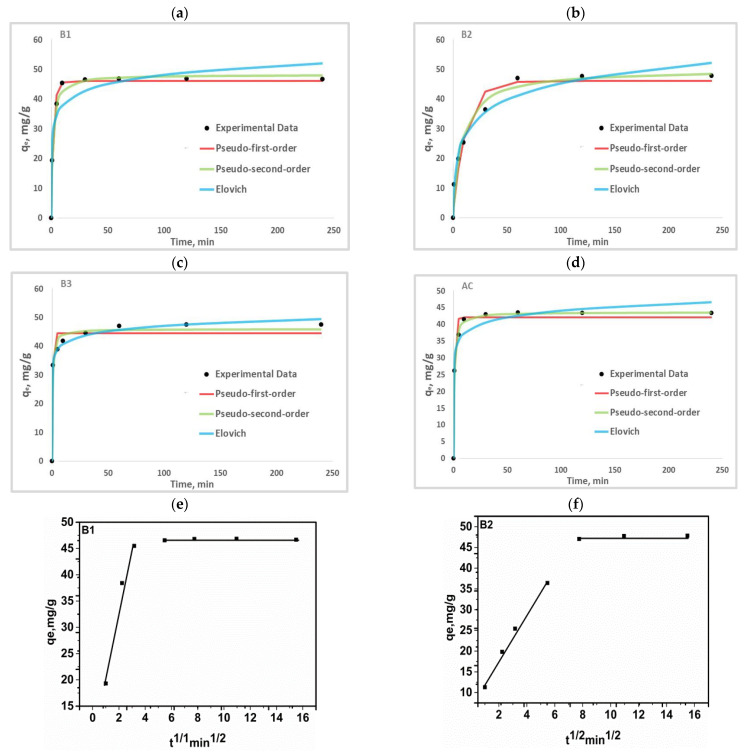

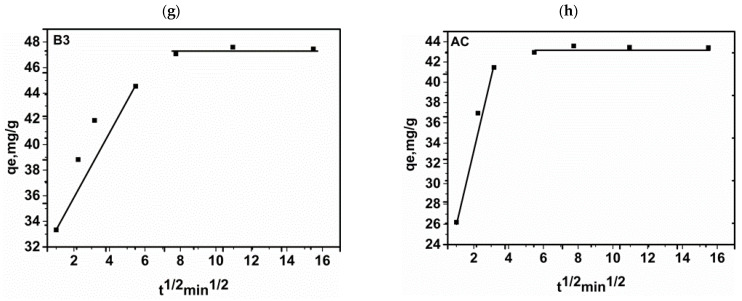
Pseudo-first order, pseudo-second order, Elovich kinetic model (**a**–**d**) and intra-particle diffusion model (**e**–**h**).

To determine the PHB adsorption mechanism on the four different adsorbents, four non-linear kinetics models were used: pseudo-first order, pseudo-second order, Elovich, and intra-particle diffusion. The fitting curves and relevant calculated parameters for all kinetic models are shown in Figure 7 and Table 3.

Higher R^2^ values correlate with lower RMSE and χ^2^ values. In comparison to other models, the pseudo-second order model best describes the kinetic data for PHB adsorption on B1, B2, and AC, while the Elovich model best describes the adsorption kinetics of B3 (Figure 7a–d). The pseudo-first order and pseudo-second order models indicate that physical and chemical adsorption play important roles in PHB retention on the studied materials [19]. In addition, the value of q_e_ calculated from the pseudo-first-order model was closer to the experimental value of q_e_. The error functions (RMSE and χ^2^) of the pseudo-second order model were very small compared to the other models, as shown in Table 3. Table 4 shows the mathematical expression of the kinetic models. The Elovich model’s initial rate constant (α) for B3 is higher than for B1 and B2, indicating that the size of our adsorbents affects the adsorption mechanism due to their specific surface area. The initial rate constant α for AC is higher than that of the biosorbents because it has a larger specific surface area; but this value is close to that of B3 which suggests that B3 has characteristics close to AC. We note that, the initial rate constant (α) is higher than the desorption rate constant, (β) which suggests that there is chemical reaction on the heterogeneous surface of the adsorbents [39].

The Weber and Morris intra-particle diffusion model was used to assess the limiting steps in PHB adsorption on the studied materials. The plot of q_e_ versus t1/2 (Figure 7) shows that PHB retention on all our adsorbents involve several steps. Mutavdžić Pavlović et al. (2021), [40] found that the emerging contaminant PHB was retained on adsorbents following three limiting steps: external diffusion of PHB from the bulk solution to the external surface of the adsorbent; internal diffusion of PHB through the pores of the adsorbent; last step achievement of the equilibrium.

Figure 7e–h shows us two linear sections with different values of slope and y-intercept. This suggests that during PHB internal diffusion, various steps could be involved. Stage 1 corresponds to adsorption by macro and mesopores, on the outer surface of the adsorbents and leads to the saturation of the latter. Once the outer layer is saturated, stage 2 begins, and the phenobarbital gradually seeps into the micropores. This is characterised by the values of the velocity constant (K_id_) and the constant (C) describing the thickness of the boundary layer in the order K_id1_ > K_id2_ and C_1_ > C_2_, respectively (Table 3); similar results were obtained by Sotelo et al. (2013) [41]. These values confirm the fact that the adsorption rate decreases progressively while the effect on the boundary layer increases during the phenomenon. The intra-particle diffusion model does not describe the adsorption mechanism of PHB on our adsorbents in view of its poor agreement between experimental and calculated data (low R^2^ values).

### 3.7. Adsorption Isotherm Studies

**Figure 8:**
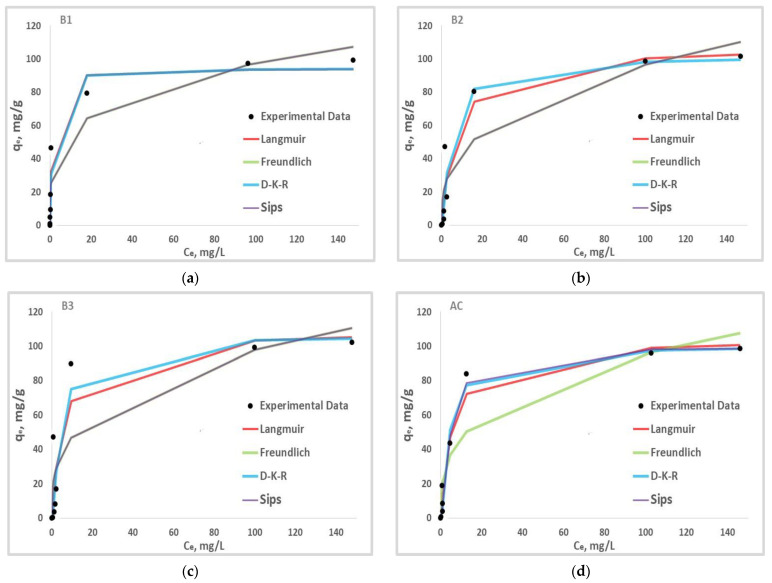
Langmuir, Freundlich, D-K-R and Sips isotherm adsorption model for B1 (**a**), B2 (**b**), B3 (**c**) and AC (**d**).

Adsorption isotherms help us understand how the PHB molecules interact with the active sites of the adsorbent [42]. To describe the behaviour of the contaminant phenobarbital on the studied adsorbents, four nonlinear isotherm models were investigated: Langmuir, Freundlich, Dubin-Kaganer-Radushkevich (D-K-R), and Sips. PHB adsorption isotherms on the four adsorbents investigated are obtained by plotting the adsorbed quantity (q_e_) against the equilibrium concentration (C_e_) for each material (Figure 8). These plots are all part of the L-type isotherm, according to the Giles table [43]. L-type isotherms, also known as Langmuir isotherms, show that the total number of adsorption sites remains constant, whereas increasing the number of adsorbed molecules reduces the number of available sites.

The values of the models’ parameters are given in Table 5 and the expressions of the models’ mathematical representations are given in Table 6. The Langmuir model describes the PHB adsorption on B1 and B2 best due to its high R^2^ values. The isotherm assumes that adsorption occurs on a monolayer with strong interactions between the adsorbate and the adsorbent. On the other hand, the fact that the separation factor R_L_ is less than one for all materials indicates that the adsorption process is favourable [44]. The Freundlich model fitting values of 0 < 1/n < 1 confirm this observation, indicating favourable adsorption [45].

The data collected from the Langmuir and Freundlich models are typically not sufficient to describe the physical or chemical nature of the adsorption process. The Dubinin–Kaganer–Radushkevich (D-K-R) isotherm is widely used to describe an adsorption mechanism with a Gaussian energy distribution over a heterogeneous surface [30].

This model is frequently used to distinguish between the physical and chemical adsorption of adsorbate molecules based on their average free energy E.

According to Table 5, given the small values of the D-K-R adsorption isotherm’s K_ad_ and the energy constant (E) for all adsorbents above 8 kJ/mol, the chemisorption with the creation of chemical bonds dominates the adsorption processes. This reinforces the Langmuir model’s premise of strong interactions between adsorbents and adsorbates. With R^2^ values close to one, the Sips isotherm model accurately represents the adsorption mechanism on adsorbents B3 and AC. This isotherm, with a value of nS (representing surface heterogeneity) other than unity, suggests that adsorption takes place on a heterogeneous surface [46].

### 3.8. Adsorption Mechanism

**Figure 9:**
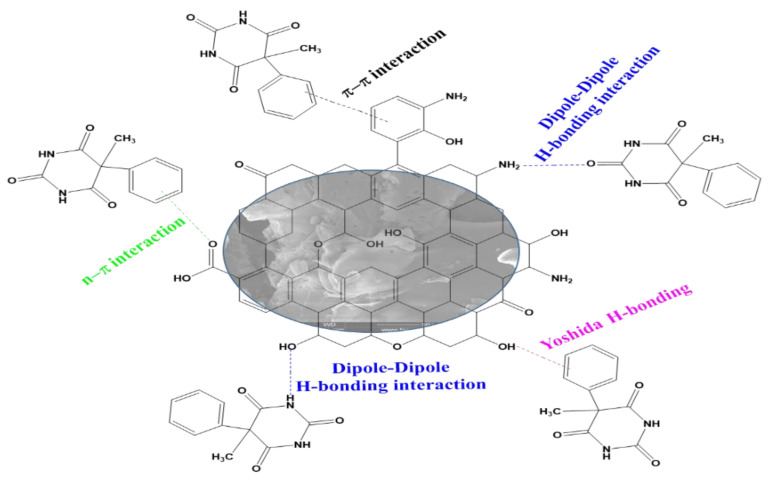
Mechanism of phenobarbital adsorption.

Figure 9 depicts the proposed mechanism for PHB adsorption onto a biosorbent. Various interactions have been proposed, including n-π, π-π, hydrogen bonding [39,47], and the Yoshida bond [48]. It is important to note that the adsorption mechanism is determined by the adsorbent’s properties (surface functional groups, surface area, contact area, and pore size distribution) as well as the adsorbate’s properties.

○ The n-π interaction studied involves the carbonyl oxygen (as electron donor) on the adsorbent surface and the aromatic rings of the organic pollutant (electron acceptor) [49].
○ According to Żółtowska-Aksamitowska et al. (2018) [50], the π-π interaction between the π electrons of the material (donor) and the π electrons of the aromatic ring of the adsorbate (acceptor) plays a minor role in the adsorption of organic materials on weak aromatic structures (biosorbents). This is in contrast to the activated carbon behaviour [51].
○ Hydrogen interactions are important factors contributing to adsorption; they involve a hydrogen atom and an electronegative atom (oxygen) [51].

The Van der Walls force is identified as a key contributor to adsorption because adsorption of organic compounds onto biosorbents via a pore-filling mechanism is less important compared to surface interactions [52].

## 4. Cost Evaluation, Economic and Environmental Analysis

The cost of developing an adsorbent for wastewater treatment is an essential concern [19,53]. This study calculated the cost of producing activated carbon and biosorbent based on the cost of power in the AD production location (Cameroon). The raw material (baobab shells) was collected for free in northern Cameroon, notably Adamaoua (Ngaoundéré). The demineralized water used for washing was obtained from the laboratory’s own facilities; the impregnation, carbonisation, washing, and drying steps, as well as the grinding stages for biosorbent production, were all considered in the cost evaluation of activated carbon production (Table 7). Given that the cost of energy in Cameroon is around 0.16 USD/kWh, the projected cost of producing 1 kg of activated carbon is 8.19 USD, and 1 kg of biosorbent is 5.82 USD.

## 5. Conclusions

This study compared the adsorption efficiency of biosorbents and activated carbon derived from baobab shells for phenobarbital removal in wastewater. SEM images revealed the presence of macroporous box set-like structures with an irregular shape. The adsorption test results revealed that, in terms of biosorbent particle size, biosorbent B3 is the best because it has the smallest particle size, promotes pollutant diffusion, and, thus, has a higher adsorption capacity. Because activated carbon has a large specific surface area and adsorption capacity comparable to B3, we conclude that B3 is an effective material, making it an affordable alternative for many other applications. EDX indicates that the biosorbent is oxygen-rich, and, thus, has several functional groups containing oxygen on its surface, as demonstrated by FTIR; Goertzen et al. (2010) [54] demonstrated that such functionalities are active groups for efficiently adsorbing pollutants. FTIR revealed the presence of several chemical functions on biosorbents that were not present on activated carbon; most notably, –C=O, which improves biosorbent performance in phenobarbital removal. The pseudo-second order and Elovich model best described the kinetic data, whereas the isothermal Sips and Langmuir models described the equilibrium data. Our materials could be used as adsorbents for wastewater treatment due to their high adsorption capacities, low cost and because they are environmentally friendly.

The adsorbents can be regenerated for reuse. This involves removing the adsorbed compounds from the surface. In future studies, we plan to investigate the regeneration of our materials and to perform experiments with real wastewater. The desorption tests will tell us if regeneration is feasible, or the materials will be disposed of by other methods (e.g., incineration).

## Figures and Tables

**Table 1:** Physico-chemical properties of phenobarbital [24].

Molecular structure	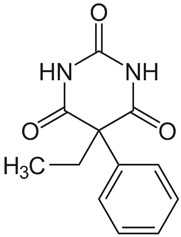
Molecular formula	C_12_H_12_N_2_O_3_ (5-ethyl-5-phenylbarbituric acid)
Molecular weight (g/mol)	232.2 g/mol
pKa	7.4
Appearance	white powder
Melting temperature	140 °C
solubility	Insoluble in water at 23 °C

**Table 2:** Values of equilibrium-adsorbed quantities.

Sample	q_e_ mg·g^−1^	Reference
B1	46.8	This work
B2	47.0	This work
B3	47.1	This work
AC	43.6	This work
Powered AC	0.41	[36]
Granular AC	0.29	[36]
PES (polyethersulfone)—AC	0.31	[36]
Garcinia kola powdered seeds	30.3	[37]
Polymeric adsorbent	2.485	[38]

**Table 3:** Parameters and fitting coefficients for kinetic models.

	Unit	B1	B2	B3	AC
q_e_(exp)	(mg∙g^−1^)	46.82	47.05	47.08	43.58
Pseudo-first order model					
q_e_(cal)	(mg∙g^−1^)	46.225	46.114	44.589	42.094
k_1_	(min^−1^)	0.452	0.086	1.366	0.939
R^2^	/	**0.992**	**0.957**	**0.963**	**0.981**
RMSE	/	1.649	4.410	3.252	2.230
χ2	/	0.610	16.643	1.424	0.719
Pseudo-second order model					
q_e_(cal)	(mg∙g^−1^)	48.254	50.099	45.971	43.706
K_2_	(mg∙g^−1^∙min^−1^)	0.016	0.002	0.049	0.033
R^2^	/	**0.994**	**0.977**	**0.985**	**0.998**
RMSE	/	1.484	3.102	2.109	0.679
χ2	/	0.352	6.735	0.631	0.073
Elovich model					
A	(mg∙g^−1^∙min^−1^)	2.22 × 10^3^	22.915	893,573.914	104,317.757
Β	(g∙min^−1^)	0.225	0.125	0.367	0.342
R^2^	/	**0.903**	**0.973**	**0.995**	**0.963**
RMSE	/	5.606	3.223	1.234	3.094
χ2	/	5.403	1.550	0.207	1.579
Intra-particle diffusion model					
Stage 1					
K_id1_	(mg∙g^−1^∙min^−1/2^)	12.28	5.54	2.39	7.14
C_1_	(mg∙g^−1^)	8.19	6.78	32.52	19.63
R^2^	/	**0.96**	**0.99**	**0.88**	**0.97**
Stage 2					
K_id2_	(mg∙g^−1^∙min^−1/2^)	0.05	0.09	0.04	0.03
C_2_	(mg∙g^−1^)	46.33	46.88	43.03	
R^2^	/	**0.75**	**0.73**	**0.39**	**0.24**

**Table 4:** Mathematical expression of kinetic models.

Kinetic Model	Mathematical Expression
Pseudo-first order	q_t_ = q_1_ (1 − e^−k1∙t^) q_1_: is the maximum amount of solute adsorbed on the solid surface by mass of adsorbent at equilibrium (mg g^−1^) q_t_: is the amount of solute adsorbed on the solid surface at time t (mg g^−1^) k_1_: is pseudo-first-order kinetic constant (min^−1^)
Pseudo-second order	q_t_ = $\frac{q_{e}^{2}k_{2}t}{1+q_{e}k_{2}t}$ q_e_: the maximum amount of solute adsorbed on the solid surface by mass of adsorbent at equilibrium (mg g^−1^) q_t_: is the amount of solute adsorbed on the solid surface at time t (mg g^−1^) k_2_: is pseudo-second order kinetic constant (mg g^−1^ min^−1^) t: contact time (min^−1^)
Intra-particle diffusion model	q_t_ = $K_{id}$t^1/2^ + C q_t_: absorbed mg g^−1^ at the t and equilibrium times K_id_: (mg g^−1^ min^−1^) is constant intraparticle difusion C: is the intercept of the plot
Elovich	q_t_ = $\frac{1}{\beta }$ ln (1 + αβt) α: initial rate constant β: desorption rate constant t: contact time (min^−1^)

**Table 5:** Langmuir, Freundlich, D-K-R and Sips isotherm adsorption parameters for PHB adsorption on B1, B2, B3 and AC.

	Unit	B1	B2	B3	AC
Langmuir model					
q_m_	(mg∙g^−1^)	94.295	107.553	109.296	104.575
K_L_	(L∙mg^−1^)	1.297	0.140	0.176	0.177
R_L_	/	0.025	0.192	0.159	0.159
R^2^	/	**0.964**	**0.922**	**0.851**	**0.973**
RMSE	/	8.529	12.895	18.420	7.412
χ2	/	17.826	60.812	128.493	21.342
Freundlich model					
K_F_	(L∙g−1)	32.131	20.134	23.354	22.818
1/n	/	0.241	0.341	0.311	0.311
R^2^	/	**0.918**	**0.846**	**0.758**	**0.864**
RMSE	/	12.899	18.109	23.497	16.753
χ2	/	47.589	81.417	124.158	60.902
D-K-R					
q_S_	(mg∙g^−1^)	94.783	101.965	107.069	100.987
K_ad_	(mol^2^∙J^−2^)	0.0003	0.001	0.001	0.001
E	(KJ∙mol^−1^)	36.863	18.393	18.637	18.686
R^2^	/	**0.958**	**0.912**	**0.839**	**0.973**
RMSE	/	9.372	13.828	19.420	7.568
χ2	/	18.860	60.623	292.607	62.350
Sips model					
q_Ms_	(mg∙g^−1^)	309,067.602	238,102.362	248,308.508	99.589
K_S_	(L∙g^−1^)	0.0001	8.453 × 10^−5^	9.40 × 10^−5^	0.109
n_S_	/	0.242	0.341	0.311	1.394
R^2^		**0.959**	**0.920**	**0.874**	**0.991**
RMSE		13.932	19.559	25.379	6.482
*χ2*		47.586	81.425	124.191	35.321

**Table 6:** Mathematical expression of adsorption isotherm models.

Isotherms Models	Mathematical Expression
Langmuir	q_e_ = $\frac{q_{m}\cdot K_{L}\cdot Ce}{1+K_{L}\cdot Ce}$ q_e_: the amount of absorbate (mg g^−1^) C_e_: absorbate equilibrium concentration (mg L^−1^) q_m_: Langmuir constant related to adsorption capacity K_L_: Langmuir constant related to energy of adsorption $R_{L}=\frac{1}{1+K_{L}C_{0}}$
Freundlich	q_e_ = K_F_·C_e_^1∕n^ q_e_: the amount of absorbate (mg g^−1^) C_e_: absorbate equilibrium concentration (mg L^−1^) K_F_: Freundlich constant related to adsorption capacity n: Freundlich constant related to adsorption intensit
D-K-R	q_e_ = q_m_$\cdot e^{-KD\varepsilon^{2}}$ q_e_: the amount of absorbate (mg g^−1^) ε: polanyi potential K_D_: free energy
Sips	q_e_ = $\frac{q_{s}(Ks\cdot {Ce}^{ns})}{1+(Ks\cdot {Ce}^{ns})}$ q_s_: is the monolayer adsorption capacity (mg g^−1^) ns: describes the adsorbent surface heterogeneity Ks: Sips isotherm constant related to energy of adsorption (L mg^−1^)

**Table 7:** Activated carbon and biosorbent production’s cost estimation.

Step	Sub-Sections	Cost Break-Up	Total Cost (USD/kg of Material)
Raw material processing	Collection of raw materials	Cost of raw material	0.0
Transportation cost			0.48
Preparation of biosorbent	Washing process	Washing cost 1 L of demineralized water corresponds to 0.16 USD, for this step we used 10 L of demineralized water. 5 × 0.16 = 0.8	0.8
Preparation of biosorbent	Drying process	Drying cost Hours × power × unit cost = 24 × 0.75 × 0.16	2.88
Preparation of biosorbent	Crushing process	Crushing cost 1 kg crushed in the machine corresponds to 1.66 USD	1.66
**TOTAL**			**5.82**
Preparation of AC	Impregnation with H_3_PO_4_	Impregnation with H_3_PO_4_ cost Hours × power × unit cost + amount of H_3_PO_4_ × unit cost = 2 × 0.75 × 1.6 + 6.3 mL × 176.26/2500 mL	2.84
Preparation of AC	Carbonization process (AC)	Carbonization cost Hours × power × unit cost = 1.37 × 1.8 × 0.16	0.39
Preparation of AC	Drying process (AC)	Drying cost Hours × power × unit cost = 24 × 0.75 × 0.16	2.88
Preparation of AC	Washing process (AC)	Washing cost 1 L of demineralized water corresponds to 0.16 USD, for this step we used 10 L of demineralized water. 10 × 0.16 = 1.6	1.6
**TOTAL**			**8.19**

## Data Availability

Data can be provided upon request.
